# Action of fibroblast growth factor-2 on the intervertebral disc

**DOI:** 10.1186/ar2407

**Published:** 2008-04-24

**Authors:** Xin Li, Howard S An, Michael Ellman, Frank Phillips, Eugene J Thonar, Daniel K Park, Ranjith K Udayakumar, Hee-Jeong Im

**Affiliations:** 1 Department of Biochemistry, Rush University Medical Center, Cohn Research BD 516, 1735 W. Harrison, Chicago, IL 60612, USA; 2 Department of Orthopedic Surgery, Rush University Medical Center, Chicago, IL 60612, USA; 3 Department of Internal Medicine, Section of Rheumatology, Rush University Medical Center, Chicago, IL 60612, USA

## Abstract

**Introduction:**

Fibroblast growth factor 2 (FGF2) is a growth factor that is immediately released after cartilage injury and plays a pivotal role in cartilage homeostasis. In human adult articular cartilage, FGF2 mediates anti-anabolic and potentially catabolic effects via the suppression of proteoglycan (PG) production along with the upregulation of matrix-degrading enzyme activity. The aim of the present study was to determine the biological effects of FGF2 in spine disc cells and to elucidate the complex biochemical pathways utilized by FGF2 in bovine intervertebral disc (IVD) cells in an attempt to further understand the pathophysiologic processes involved in disc degeneration.

**Methods:**

We studied the effect of FGF2 on IVD tissue homeostasis by assessing MMP-13 expression (potent matrix-degrading enzyme), PG accumulation, and PG synthesis in the bovine spine IVD, as well as evaluating whether FGF2 counteracts known anabolic factors such as BMP7. To understand the molecular mechanisms by which FGF2 antagonizes BMP7 activity, we also investigated the signaling pathways utilized by FGF2 in bovine disc tissue.

**Results:**

The primary receptor expressed in bovine nucleus pulposus cartilage is FGFR1, and this receptor is upregulated in degenerative human IVD tissue compared with normal IVD tissue. Stimulation of bovine nucleus pulposus cells cultured in monolayer with FGF2 augmented the production of MMP-13 at the transcriptional and translational level in a dose-dependent manner. Stimulation of bovine nucleus pulposus cells cultured in alginate beads for 21 days with FGF2 resulted in a dose-dependent decrease in PG accumulation, due at least in part to the inhibition of PG synthesis. Further studies demonstrate that FGF2 (10 ng/ml) antagonizes BMP7-mediated acceleration of PG production in bovine nucleus pulposus cells via the upregulation of noggin, an inhibitor of the transforming growth factor beta/bone morphogenetic protein signaling pathway. Chemical inhibitor studies showed that FGF2 utilizes the mitogen-activated protein kinase and NF-κB pathways to upregulate noggin, serving as one potential mechanism for its anti-anabolic effects.

**Conclusion:**

FGF2 is anti-anabolic in bovine spine disc cells, revealing the potential of FGF2 antagonists as unique biologic treatments for both prevention and reversal of IVD degeneration.

## Introduction

Back pain is a common ailment among American adults, with a lifetime prevalence of approximately 70% to 85% in the United States [1]. While the etiology is largely unknown, the pathological degeneration of the intervertebral disc (IVD) has been associated with chronic back pain [2,3]. At present, the current treatments for back pain are mainly symptomatic or involve surgical procedures that ablate the disc, but most strategies make no attempt to interfere with early biochemical and pathophysiologic processes involved in disc degeneration. Elucidation of the contributory metabolic pathways at play would therefore enable us to focus on more specific treatment regimens in the future.

Structurally, the IVD consists of tough outer rings, collectively termed the annulus fibrosus (AF), and a gelatinous inner core, the nucleus pulposus (NP). This unique structure has both shock-absorbing properties and the ability to resist deformation upon mechanical loading. The AF is composed mainly of collagen secreted by disc cells, while the NP is largely composed of proteoglycans (PGs), principally aggrecan. It has been suggested that the degenerative process begins in the NP and is associated with the progressive loss of PGs [2].

Disc cells residing in both the AF and NP actively regulate matrix homeostasis through activities modulated by a variety of stimuli, including cytokines and growth factors acting in a paracrine and/or autocrine fashion. The cells in the normal adult IVD maintain the matrix in which they reside at a steady state. Degeneration of the IVD may result from an imbalance between the anabolic and catabolic processes and loss of this steady-state metabolism [4]. IVD damage caused by mechanical injury, inflammation, or aging may change the structure of the IVD, shifting IVD homeostasis and disc cell-mediated gene expression in favor of a procatabolic state. Evidence shows that matrix metalloproteases (for example, MMP-13 – otherwise known as collagenase 3) and aggrecanases (ADAMTS4 and ADAMTS5) – enzymes strongly upregulated by proinflammatory cytokines – may have critical pathogenic roles in the extracellular matrix (ECM) degradation that characterizes the degeneration of the IVD [5]. In particular, MMP-13 has been shown to act as a PG-degrading enzyme in addition to assisting in collagen degradation, and thus may play a dual role in IVD degeneration [6].

Regenerative medicine is aimed at regulating the metabolism of IVD cells to achieve biological regeneration that will have more permanent therapeutic benefits than synthetic or metallic implants. Anabolic regulators of IVD homeostasis include polypeptide growth factors, such as insulin-like growth factor 1, transforming growth factor beta (TGFβ) and the bone morphogenetic proteins (BMPs) [7]. In particular, numerous reports have implied the anabolic effect mediated by BMP7 (otherwise known as osteogenic protein-1) on cartilage regeneration in both articular joints [8] and spine discs *in vitro *[9,10]. Catabolic regulators of IVD homeostasis, on the other hand, include proinflammatory cytokines and growth factors such as IL-1 [5,11,12] – and potentially fibroblast growth factor 2 (FGF2) (otherwise known as basic fibroblast growth factor) [13] – both of which have been implicated in the degeneration of the IVD. An upregulation of anabolic factors coupled with a downregulation of catabolic factors may potentially induce cartilage regeneration.

In cartilage, FGF2 is produced by chondrocytes, is stored in the ECM, and is immediately released from the ECM upon cartilage injury [14]. We recently reported significant upregulation of FGF2 and its cognate receptor, fibroblast growth factor receptor type I (FGFR1), in arthritic articular cartilage compared with normal cartilage [13]. In human adult articular cartilage, FGF2 stimulates cartilage-degrading enzyme expression, inhibits PG accumulation and synthesis, and antagonizes the anabolic activity of insulin-like growth factor 1 and BMP7, suggesting that FGF2 plays a principal pathophysiological role in articular cartilage [8,13,15,16]. In the IVD, Peng and colleagues demonstrated highly upregulated FGF2 and FGFR1 in painful degenerated human spine disc cells compared with normal cells [17]. Further immunohistologic studies have demonstrated the presence of FGF2 in human herniated IVD tissue [18,19] and in injured AF tissue in adult merinos [20]. While these findings demonstrate the localization and/or expression of FGF2 in IVD tissue, however, the function and biological effects mediated by FGF2 in spine discs have yet to be assessed.

In the current study, we determined the role of FGF2 in the IVD using bovine disc cells. Specifically, we studied the effect of FGF2 on IVD homeostasis by assessing MMP-13 production, PG accumulation, and PG synthesis in the bovine spine, as well as evaluating whether FGF2 counteracts known anabolic factors such as BMP7. Our results may provide important new information on spine disc metabolism mediated by FGF2 relative to the understanding of IVD degeneration as one mechanism of low back pain.

## Materials and methods

### Nucleus pulposus and annulus fibrosus cell isolation and culture

Human lumbar IVDs were obtained from cadaveric donor spines (Gift of Hope) from June 2004 to June 2005. The gross morphology of each disc was graded by the Thompson grading scheme [21] after magnetic resonance imaging T2 imaging. NP tissue from normal discs (grade 0 to 2) and from degenerative discs (grade 3 to 5) was separated from the AF tissue. Cells were released by enzymatic digestion, as previously described [22], and were analyzed using RT-PCR as described below. The experiments were repeated twice, using discs from two cadaveric spines.

Bovine IVD tissue was obtained from bovine tails of young adult animals (15 to 18 months old, purchased from a local slaughterhouse). Coccygeal discs were opened *en bloc*, and the NP and AF portions of each disc were separated. The cells were released by enzymatic digestion in DMEM/Ham's F-12 (1:1) culture medium with sequential treatments of 0.2% pronase and 0.025% collagenase P, as previously described [23]. Alginate beads and monolayers were made for long-term and short-term analysis, respectively.

For alginate bead cultures, isolated NP cells and AF cells were resuspended in 1.2% alginate, and beads were formed by dropwise addition into a CaCl_2 _solution, as previously described [24]. Briefly, beads were cultured at eight beads per well in 24-well plates in 1 ml/well DMEM/Ham's F-12 medium (1/1) supplemented with 1% mini-insulin–transferrin–selenium [23,25]. Cells were treated with 0.1, 0.5, 1, 5, and 10 ng/ml FGF2 (NCI, Bethesda, MD, USA), 1 ng/ml IL-1β (Amgen, Thousand Oaks, CA, USA) for catabolic control, or 100 ng/ml BMP7 (Stryker Biotech, Hopkinton, MA, USA) for anabolic control. Triplicate wells were used for each condition. Media was changed every other day for a 21-day period before dimethylethylene blue (DMMB) analysis.

For monolayer cultures, isolated NP cells were counted and plated at 8 × 10^5 ^cells/cm^2 ^as previously described [8,13]. For supernatant analysis, cells were treated with FGF2 (0, 0.5, 5, and 10 ng/ml) or with FGF18 (10 ng/ml; PeproTech, Rocky Hill, NJ, USA), and the supernatant was removed 24 hours after the addition of treatments and subjected to immunoblotting with anti-MMP-13 antibody, which can recognize the pro-form and activated form of MMP-13 (R&D Systems, Minneapolis, MN, USA). For gene expression analysis, NP cells harvested after treatment with FGF2 or FGF18 were analyzed for MMP-13, ADAMTS4, and ADAMTS5 mRNA expression using RT-PCR, as described below. In addition, NP cells cultured in monolayer were treated with FGF2 for 24 hours in the presence of ERK inhibitor (PD98059, 25 μM; Calbiochem, Gibbstown, NJ, USA) or IKK inhibitor (Wedelolactone, 2.5 μM; Calbiochem), and were subjected to RT-PCR for analysis of noggin (an inhibitor of TGFβ/bone morphogenetic protein signaling pathway) gene expression. Control NP cells (no treatment) were analyzed for FGFR1 to FGFR4 mRNA expression.

### Immunoblotting

The total protein concentrations of media were determined by a bicinchoninic acid protein assay (Pierce, Rockford, IL, USA). In each case, an equal amount of protein was resolved by 10% SDS-polyacrylamide gels and transferred to nitrocellulose membrane for immunoblot analyses as described previously [13]. Immunoreactivity was visualized using the ECL system (Amersham Biosciences, Piscataway, NJ, USA) and the Signal Visual Enhancer system (Pierce), which magnifies the signal.

### Reverse transcription and real-time polymerase chain reaction

Total cellular RNA was isolated using the Trizol reagent (Invitrogen, Carlsbad, CA, USA) following the instructions provided by the manufacturer. Reverse transcription was carried out with 1 μg total cellular RNA using the ThermoScript™ RT-PCR system (Invitrogen) for first-strand cDNA synthesis in 50 μg reaction volume.

For semiquantitative PCR, each reverse transcription sample was assessed for glyceraldehyde 3-phosphate dehydrogenase cDNA. The cDNA was amplified by PCR using 24 to 32 cycles of 95°C for 30 seconds, 55°C to 60°C for 30 seconds, and 72°C for 30 seconds in the presence of Taq polymerase (Invitrogen), 50 pmol sense and antisense primers. PCR products were resolved on 1.5% agarose gels and were visualized by staining with ethidium bromide and UV transillumination. Integrated density values for the genes in question were normalized to the glyceraldehyde 3-phosphate dehydrogenase values to yield a semiquantitative assessment.

For real-time PCR the cDNA was amplified using the MyiQ Real-Time PCR Detection System (Bio-Rad, Hercules, CA, USA). The reverse transcription product was subjected to real-time PCR in a 20 μl total reaction mixture containing 10 μl Bio-Rad iQ™ SYBR Green supermix (Bio-Rad), 1 μl of 10 μM sense and antisense primers, and 1 μl template cDNA. A threshold cycle (C_T _value) was obtained from each amplification curve using iQ5 Optical System Software provided by the manufacturer (Bio-Rad). Relative mRNA expression was determined using the ^ΔΔ^C_T _method, as detailed by manufacturer guidelines (Bio-Rad). Glyceraldehyde 3-phosphate dehydrogenase was used as the internal control in the reaction for normalization. The primer sequences and their conditions for use are summarized in Table 1.

**Table 1:** Primer sequences for RT-PCR

Gene	Primer sequence (forward/reverse) (5' to 3')	Size (base pairs)	Annealing temperature (°C)	Reference accession number
h-FGFR1	AAC CCC AGC CAC AAC, CCA AAG CTG GGC TGG GTG TCG	687	60	EMBL: NM_015850.2
h-FGF2	GAG AAG AGC GAC CCT CAC A TAG CTT TCT GCC CAG GTC C	278	58	EMBL: NM_002006.3
h-ADAMTS5	CCC TCT TCC CTG TGC AGT AG CTA CGA TGC CAC CCA GCA G	363	58	EMBL: NM_007038.2
h-MMP-13	GGC TCC GAG AAA TGC AGT CTT TCT T ATC AAA TGG GTA GAA GTC GCC ATG C	338	0	EMBL: NM_007038.2
h-GAPDH	GGT ATC GTG GAA GGA CTC AT ACC ACC TGG TGC TCA GTG TA	340	55	EMBL: XR_018317.1
Bov-MMP-13	ACC CTT CCT TAT CCC TTG ATG CCA AAA CAG CTC TGC TTC AAC CTG CTG	110	55	EMBL: NM_174389.2
Bov-ADAMTS 4	ACT GGG CTA CTA TTA CGT GGA AAA CAC ACA CCA TGC ACT TGTCGA ACT	155	60	EMBL: BC148059.1
Bov-ADAMTS 5	ACG TGG TGT TCT CTC CAA AG CAT ACT GCA GCT TCG AGC CA	146	55	EMBL: XM_589193.2
Bov-FGFR1	AGG TAA CAA GAA GAC AAG CGG GCA ATG GGC CAG TAA GTG AAG ACC ACT	127	55	EMBL: XM_001255761.1
Bov-FGFR2	ATA CCT GCG TGG TGG AGA ACG ATT TTT GCA GAC AAA CTC CAC ATC GCC	152	55	EMBL: XM_880481.2
Bov-FGFR3	GTG GCC GTG AAG ATG CTG AAA GAT AGG CGC CTA GCA GGT TGA TAA TGT	120	55	EMBL: NM_174318.2
Bov-FGFR4	GCT GAT TGG CCG ACA CAA GAA CAT AGC ACA CTC CAC GAT CAC GTA CAA	85	55	EMBL: XM_602166.3
Bov-noggin	TCT GTA ACT TCC TCC GCA GCT TCT AGC GAG ATC AAA GCG CTG GAG TT	88	55	EMBL: XM_582573.3
Bov-β-actin	AAG AGA TCA ATG ACC TGG CAC CCA ACT CCT GCT TGC TGA TCC ACA TCT	141	55	EMBL: BT030480.1

ADAMTS = a disintegrin and metalloproteinase with thrombospondin motifs; FGF2 = fibroblast growth factor 2; FGFR = fibroblast growth factor receptor; GAPDH = glyceraldehyde 3-phosphate dehydrogenase; MMP = matrix metalloprotease.

### Dimethylethylene blue assay for proteoglycan production and DNA assay for cell numbers

At the end of the 21-day alginate culture period the medium was removed, and the alginate beads were collected and processed for PG assays using the DMMB binding method, as previously described [25]. The cell-associated matrix (CM) was separated from the further-removed matrix, and PG accumulation per cell in the CM was quantified [25]. Cell numbers were determined by assay of total DNA in the cell pellets using PicoGreen (Molecular Probes, Carlsbad, CA, USA), as previously described [23].

### [^35^S]-Sulfate incorporation into newly synthesized proteoglycans

The same labeling protocol was used for all cultures. On day 7 of culture in alginate, the medium was removed and replaced by fresh medium. One hour later, this medium was replaced with fresh medium containing [^35^S]-sulfate at 20 μCi/ml (Amersham Corp, Arlington Heights, IL, USA). After incubation for 4 hours, the labeling medium was removed and the beads were rinsed twice in cold 1.5 mM SO_4 _wash media. Beads were dissolved to separate out the CM and were digested with papain (20 μg/ml in 0.1 M sodium acetate, 0.05 M ethylenediamine tetraacetic acid, pH 5.53) at 60°C for 16 hours. Sulfate incorporation into PGs was measured using the Alcian blue precipitation method [26]. All samples were analyzed in duplicate and were normalized for DNA content using Hoechst 33258 as previously described [26].

### Particle exclusion assay for matrix assessment

The cells with their pericellular matrix were visualized using the particle exclusion assay, as previously described [24,27]. Briefly, after day 21 of culture in alginate, the beads were solubilized with sodium citrate. The cells were pelleted by centrifugation, resuspended in DMEM, and then placed in the bottom of a multiwell plate. The cells were allowed to settle and attach to the plates for 6 to 12 hours, and formalin-fixed erythrocytes were then added and allowed to settle for 10 to 15 minutes. Cells were then observed and photographed with an inverted phase-contrast microscope (Nikon, Melville, NY, USA).

### Statistical analysis

Analysis of variance was performed using StatView 5.0 software (SAS Institute, Cary, NC, USA). *P *< 0.05 was considered significant.

## Results

### Comparison of endogenous gene expression by cells from normal and degenerative human IVD

**Figure 1:**
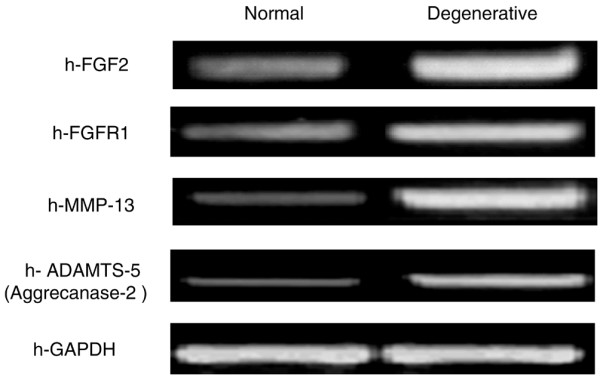
**Comparison of endogenous gene expression by cells from normal and degenerative human intervertebral disc**. Fresh human nucleus pulposus tissue from normal (grades 0 to 2) and degenerative (after surgery) intervertebral disc cells were subjected to total RNA preparation followed by semiquantitative RT-PCR using human specific primer sets. Glyceraldehyde 3-phosphate dehydrogenase (GAPDH) was used as the internal control. FGF2, fibroblast growth factor 2; FGFR, fibroblast growth factor receptor.

Fresh human NP tissue from normal IVD cells (grades 0 to 2) and degenerative IVD cells (after surgery) were subjected to total RNA preparation followed by semiquantitative RT-PCR using human specific primer sets. Our RT-PCR results demonstrated that the expression levels of mRNA for FGF2 and its cognate receptor FGFR1, as well as those for matrix-degrading enzymes MMP-13 and ADAMTS5 (also known as aggrecanase 2), are highly upregulated in degenerative human NP cells. There was no significant difference in the expression of mRNA for glyceraldehyde 3-phosphate dehydrogenase, an internal control, by the cells from degenerative and normal IVD (Figure 1). These results suggest that FGF2 and its receptor FGFR1, along with specific matrix-degrading enzymes, may play a pathogenic role in degenerative processes that accompany the loss of IVD matrix homeostasis.

### FGFR1 expression is upregulated in normal bovine nucleus pulposus tissue

**Figure 2:**
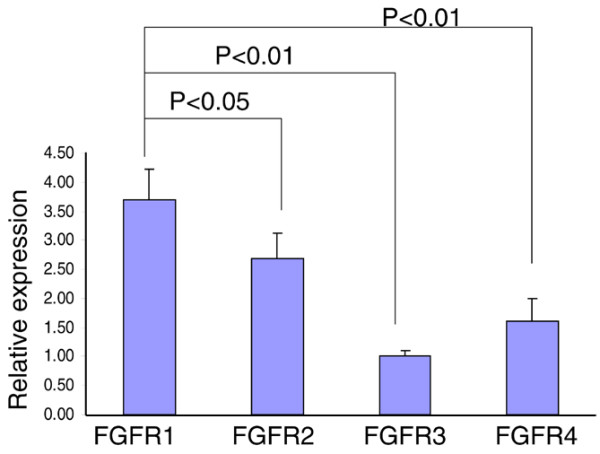
**Fibroblast growth factor receptor 1 expression is upregulated in normal bovine nucleus pulposus tissue**. Nucleus pulposus cells isolated from bovine intervertebral disc were cultured in a monolayer in 12-well plates at 8 × 10^5 ^cells/cm^2 ^for 48 hours and the total RNA was extracted to perform real-time RT-PCR of fibroblast growth factor receptor (FGFR1, FGFR2, FGFR3 and FGFR4) genes. Error bars represent three different donors in three separate experiments.

The biological activity of FGF2 is mediated through extracellular binding to its high-affinity cell surface tyrosine kinase receptors (FGFR1 to FRFR4) [28,29]. In our laboratory, we have previously found that FGFR1 and FGFR3 are highly expressed relative to FGFR2 and FGFR4 in normal human adult articular chondrocytes using flow cytometry analysis with antibodies to FGFR1 to FGFR4 (human knee cartilage; Muddasani P, Zhao LJ, Im HJ, et al, unpublished data). We therefore sought to determine the primary receptor expressed in bovine NP tissue. Based on real-time PCR results, we found that FGFR1, followed respectively by FGFR2, FGFR4, and FGFR3, is the most abundant receptor present in bovine NP tissue (Figure 2). FGFR1 was roughly 3.8 times as prevalent as FGFR3, while FGFR2 was roughly 2.8 times as prevalent as FGFR3.

### FGF2 increases the expression of cartilage-degrading enzymes by bovine intervertebral disc cells

**Figure 3:**
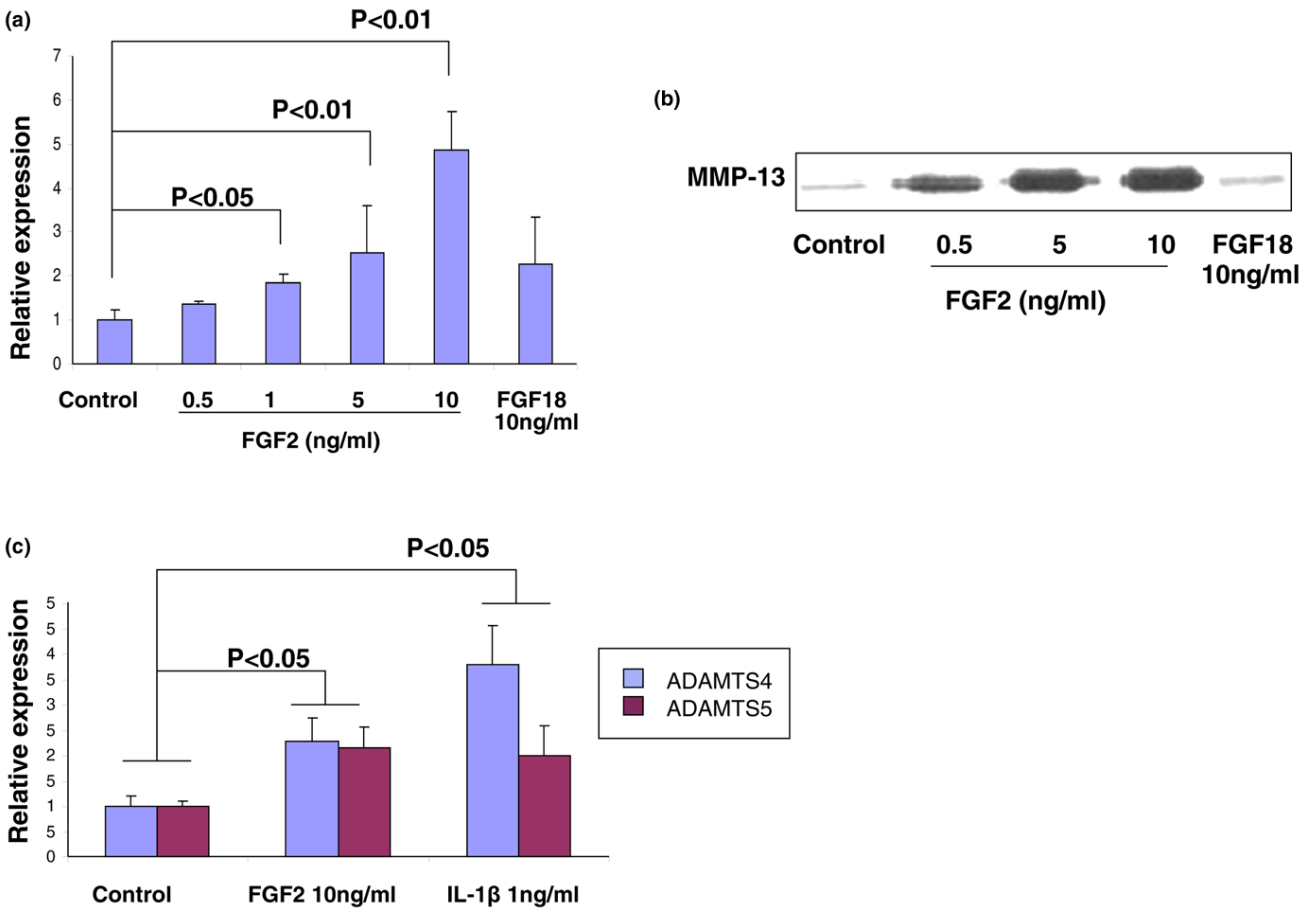
**Fibroblast growth factor 2 increases the expression of cartilage-degrading enzymes by bovine intervertebral disc cells**. Nucleus pulposus cells isolated from bovine intervertebral disc were cultured in monolayer in 12-well plates at 8 × 10^5 ^cells/cm^2^, and were serum-starved by changing the media to serum-free DMEM/F-12 with antibiotics for 24 hours before treatment. Cells were then treated with 0.1 to 10 ng/ml fibroblast growth factor 2 (FGF2) and 100 ng/ml fibroblast growth factor 18 (FGF18), collected after 24 hours, and the total RNA extracted to perform real-time RT-PCR for **(a) **MMP-13 gene expression and **(c) **ADAMTS4 and ADAMTS5 gene expression. **(b) **Conditioned media was subjected to immunoblotting for the product of pro MMP-13. Error bars represent three different donors in three separate experiments.

Recent studies have demonstrated that FGF2 stimulates the production of MMP-13 and pro-inflammatory cytokines in human adult articular cartilage [13,15,16]. We therefore tested whether FGF2 exerts similar biological activity on IVD cells. Real-time PCR results demonstrated that treatment of NP cells cultured in monolayer with FGF2 for 24 hours stimulated MMP-13 expression in a dose-dependent manner (Figure 3a). At concentrations of 1 and 10 ng/ml FGF2, MMP-13 mRNA expression increased by a factor of two and five, respectively, compared with control (untreated). In contrast, coincubation of cells with FGF18 (10 ng/ml), a member of the FGF superfamily, showed no induction of MMP-13 mRNA expression.

Western blot analysis (Figure 3b) supported these observations on the protein level, revealing an FGF2-stimulated, dose-dependent increase in the expression of the pro-form of MMP-13 compared with control, coupled with no induction of MMP-13 after stimulation with FGF18. Finally, FGF2 increased the expression of ADAMTS4 and ADAMTS5, well-known aggrecanases involved in PG degradation (Figure 3c).

### FGF2 inhibits proteoglycan accumulation in the cell-associated matrix

**Figure 4:**
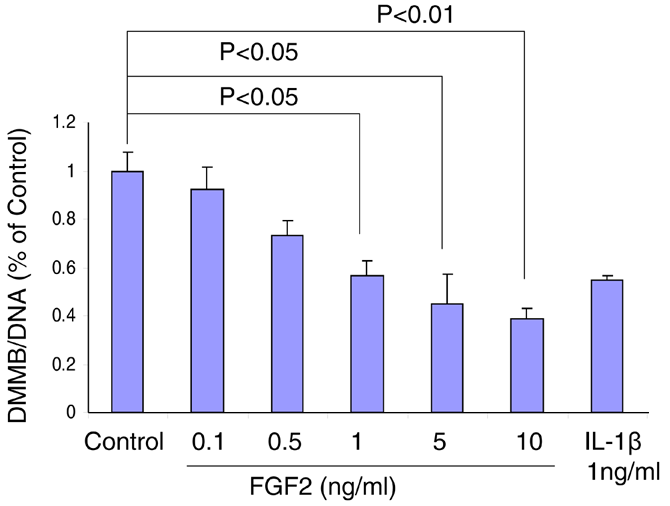
**Fibroblast growth factor 2 inhibits proteoglycan accumulation in the cell-associated matrix**. Nucleus pulposus cells isolated from bovine intervertebral disc were cultured for 21 days in 1.2% alginate beads in serum-free medium with mini-insulin–transferrin–selenium (control) or the control medium plus 0.1 to 10 ng/ml fibroblast growth factor 2 (FGF2). Control medium plus 1 ng/ml IL-1β was used as a positive control. At the end of the culture period, the beads were dissolved in sodium citrate and cell pellets were separated by centrifugation. The amount of proteoglycan in the cell-associated matrix around the cells was measured by dimethylethylene blue assay and normalized to cell numbers using DNA measurement (DMMB/DNA). Samples were measured in triplicate and expressed as a percentage of the day 21 control cultures. Error bars represent three different donors in three separate experiments.

Aggrecan, a major component of PGs, is a substrate of both aggrecanases (ADAMTS4 and ADAMTS5) and matrix metalloproteases, such as MMP-13 [16] – proteases whose production is upregulated by FGF2 (Figure 3a,c). To determine what effect FGF2 has on PG accumulation in the CM of bovine IVD cells, NP cells encapsulated in three-dimensional alginate beads were cultured for 21 days in the presence of 0.1 to 10 ng/ml FGF2 or 1 ng/ml IL-1β (Figure 4). After 21 days, the addition of 0.5 ng/ml FGF2 reduced the PG accumulation per cell to roughly 80% of control (untreated, lane 2). This effect was dose dependent, as higher concentrations of FGF2 (0.5, 1, 5 and 10 ng/ml) decreased PG accumulation per cell (80%, 55%, 45% and <45% PG accumulation compared with control, respectively). IL-1β, a cytokine with well-documented inhibitory effects on PG synthesis, was used as a negative control. At a concentration of 1 ng/ml FGF2, the total amount of PG was lower than in cells treated with IL-1β1 ng/ml. These results show that FGF2 decreases PG accumulation in the CM over 21 days of culture in a dose-dependent manner.

### FGF2-mediated reduction in proteoglycan accumulation in the cell-associated matrix

**Figure 5:**
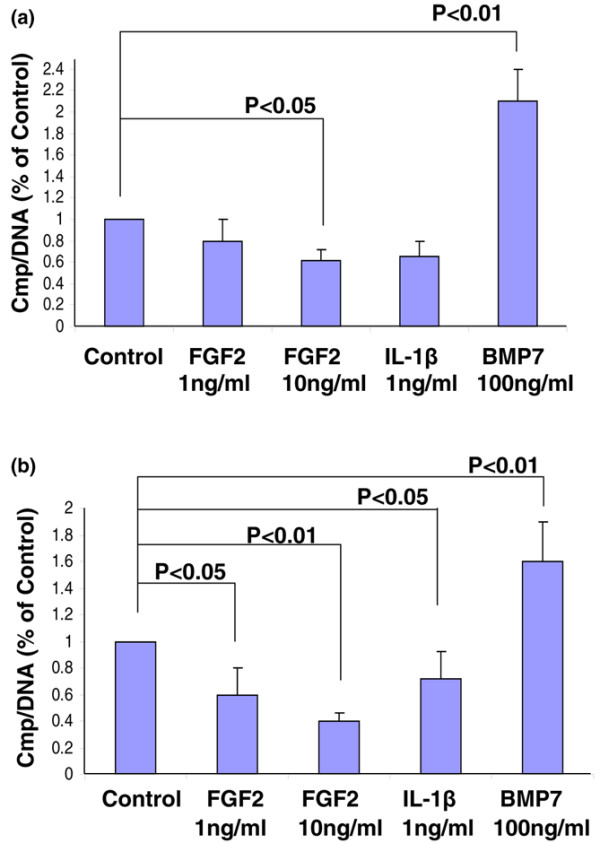
**Fibroblast growth factor 2 inhibits proteoglycan synthesis in the cell-associated matrix**. **(a) **Nucleus pulposus cells and **(b) **annulus fibrosus cells isolated from bovine intervertebral disc were cultured for 7 days in 1.2% alginate in serum-free medium with mini-insulin–transferrin–selenium (control) or the control medium plus 1 and 10 ng/ml fibroblast growth factor 2 (FGF2), 1 ng/ml IL-1β, or 100 ng/ml BMP7. Proteoglycan synthesis was measured during the last 4 hours of culture using [^35^S]-sulfate incorporation and was normalized to cell numbers by DNA assay. Data expressed as a percentage of control for triplicate samples. Error bars represent the triplicate analysis of three pooled donors.

To determine whether the reduction in PG accumulation was mediated by an FGF2-mediated inhibition of PG synthesis, the incorporation of [^35^S]-sulfate by NP and AF cells into PGs was quantified. The results showed that PG synthesis by both NP cells (Figure 5a) and AF cells (Figure 5b) was indeed suppressed in the presence of FGF2. When expressed per microgram of DNA, this inhibition was found to be dose-dependent in both cell types. IL-1β and BMP7 (a growth factor well known for its ability to promote PG synthesis by chondrocytes) were used as negative control and positive control, respectively. Interestingly, the AF cells were less responsive than the NP cells to treatment with BMP7, a finding consistent with that of previous studies [30]. Treatment with 100 ng/ml BMP7 increased PG synthesis by AF cells to 152% of control, compared with 210% of control in the case of NP cells. In bovine NP cells, treatment with 10 ng/ml FGF2 alone significantly inhibited PG synthesis, reducing the amount of PG synthesized per cell by 40%. A similar finding was noted in the case of AF cells. FGF2-mediated reduction in PG accumulation in the CM is therefore, in part, the result of an inhibition of proteoglycan synthesis.

### FGF2 antagonizes BMP7-mediated stimulation of proteoglycan accumulation

**Figure 6:**
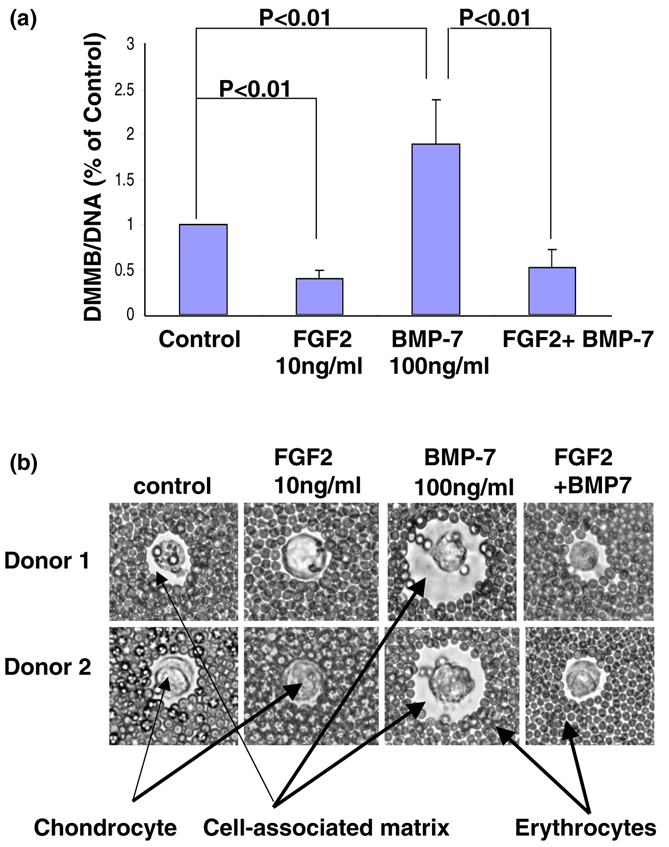
**Fibroblast growth factor 2 antagonizes BMP7-mediated stimulation of proteoglycan accumulation in the cell-associated matrix**. **(a) **Nucleus pulposus cells isolated from bovine intervertebral disc were cultured for 21 days in 1.2% alginate beads in serum-free medium with mini-insulin–transferrin–selenium (control) or the control medium plus 10 ng/ml fibroblast growth factor 2 (FGF2), 100 ng/ml BMP7, or 10 ng/ml FGF2 combined with 100 ng/ml BMP7. At the end of the culture period the beads were dissolved in sodium citrate, and cell pellets containing the cells and their cell-associated matrix (CM) were separated by centrifugation. The amount of proteoglycan in the CM was measured by dimethylethylene blue assay and normalized to cell numbers using DNA measurement (DMMB/DNA). Samples were measured in triplicate and expressed as a percentage of the day 21 control cultures. Error bars represent three different donors in three separate experiments (Fig 6A). **(b) **Nucleus pulposus cell pericellular matrix production after alginate culture for 21 days in the presence or absence of FGF2, BMP7 or the combination of both factors was measured in an exclusion assay as described in Materials and methods. A representative sample was photographed using an inverted phase-contrast microscope. The CM can be seen excluding the erythrocytes from the cell plasma membrane (original magnification × 400).

Having previously shown that FGF2 has a potent antagonistic effect on both BMP7 and insulin-like growth factor 1 in human adult articular cartilage [15], we set out to determine whether FGF2 exerts a similar biological impact on NP cells cultured in the presence of BMP7. Our results indicate that FGF2 (10 ng/ml), when present, completely abolishes the stimulation of PG accumulation by BMP7 (100 ng/ml) (Figure 6a). In the present study, BMP7 (100 ng/ml), when given alone, led to a 190% increase in PG production. When FGF2 was incorporated into the medium with BMP7, however, this anabolic effect was abolished; in fact, PG production decreased by approximately 40% compared with control. The FGF2-mediated antagonistic biological effect on BMP7 was further visualized using an exclusion assay (Figure 6b). Taken together, the results suggest that the response of bovine NP cells to exposure to FGF2 and BMP7 is very similar to that reported by Loeser and colleagues using human adult articular cartilage [15].

### FGF2 stimulates noggin via the ERK mitogen-activated protein kinase and NF-κB pathways

While previous studies have examined the antagonistic relationship between FGF2 and BMP7 [15], few have defined the molecular mechanisms or signaling cascades by which FGF2 exerts this effect. We incubated bovine NP cells in a monolayer in medium containing FGF2 at different concentrations (0.1, 1, 5 and 10 ng/ml). As IL-1β also antagonizes the matrix-producing action of BMP7, we included it in this experimental set as a control.

**Figure 7:**
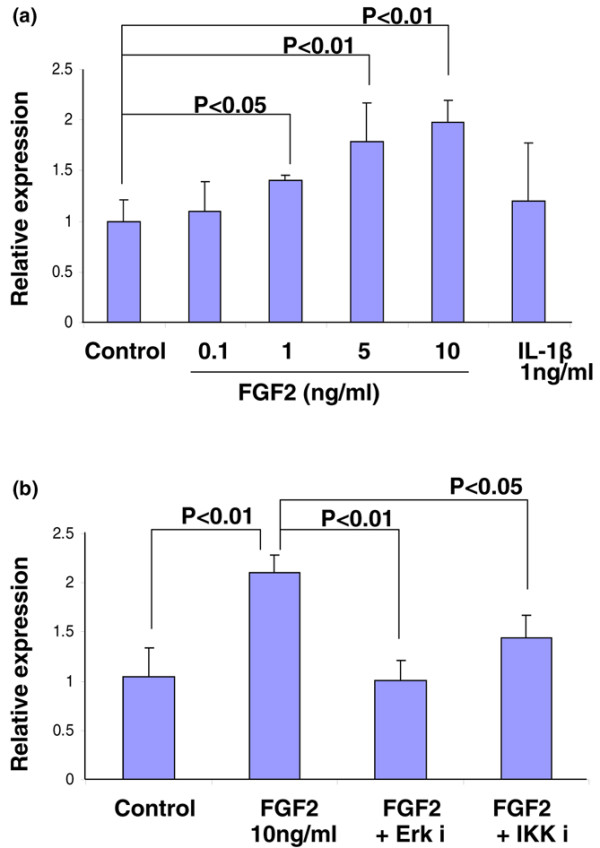
**Fibroblast growth factor 2 stimulates noggin via the ERK mitogen-activated protein kinase and NF-κB pathways**. Nucleus pulposus cells isolated from bovine intervertebral disc were cultured in a monolayer in 12-well plates at 8 × 10^5 ^cells/cm^2^, and were serum-starved by changing the media to serum-free DMEM/F-12 with antibiotics for 24 hours before treatment. **(a) **Cells were then treated with 0.1 to 10 ng/ml fibroblast growth factor 2 (FGF2) and 1 ng/ml IL-1β. **(b) **Serum-starved cells were treated with 10 ng/ml FGF2 in the presence or absence of the chemical inhibitors of ERK (ERKi, 25 μM) or IKK (IKKi, 25 μM). The cells were collected after 24 hours, and total RNA was extracted to perform real-time RT-PCR of the noggin gene. Error bars represent three different donors in three separate experiments.

Using real-time PCR, we found that stimulation of cells with FGF2 dose-dependently increased the expression of noggin, a known inhibitor of TGFβ/bone morphogenetic protein [31], presumably leading to decreased BMP7 activity (Figure 7a). FGF2 at a concentration as low as 1 ng/ml was sufficient to significantly increase (*P *< 0.05) noggin expression. Interestingly, this FGF2-mediated stimulation of noggin expression was completely neutralized upon the addition of the mitogen-activated protein kinase ERK pathway-specific inhibitor, decreasing the noggin level to that of the control group (Figure 7Bb). Moreover, giving an inhibitor of the NF-κB pathway (IKK inhibitor peptide) along with FGF2 diminished the stimulatory effect of FGF2 on noggin expression, but did not totally obliterate the effect.

Our data suggest that FGF2 activation of the ERK mitogen-activated protein kinase and NF-κB pathways are involved in the inhibitory action of FGF2 on BMP7 signaling via activation of noggin.

## Discussion

The present study demonstrates the potent anti-anabolic effects of FGF2 on IVD homeostasis. Stimulation with FGF2 mediated a dose-dependent upregulation of MMP-13, a significant inhibitory effect on PG accumulation and synthesis, and the inability of BMP7 to stimulate PG production in the presence of FGF2. In addition, the chemical pathways utilized by FGF2 to antagonize the activity of BMP7 were analyzed to gain a better understanding of the complex interplay of growth factors, cytokines, and enzymes in the IVD. To our knowledge, this is the first study that demonstrates the pathophysiologic effects of FGF2 in spine disc tissue.

Based on our DMMB results, treatment with FGF2 in alginate culture for 21 days dose-dependently decreased the accumulation of PG in NP cells. This reduction could be due to either increased PG degradation or decreased synthesis, or due to both. Examples of increased PG degradation include the FGF2-stimulated, MMP-13-mediated or ADAMTS4-mediated and ADAMTS5-mediated destruction of aggrecan. Accumulated evidence has indicated that in arthritic articular cartilage the overproduction of collagenases, in particular MMP-13, by chondrocytes plays a central role in collagen and aggrecan degradation [5,32-34]. We found that FGF2, MMP-13, and ADAMTS5 were upregulated in human degenerative disc tissue compared with normal discs (Figure 1), and that FGF2 stimulated ADAMTS4 and ADAMTS5 expression, as well as a dose-dependent increase in MMP-13 expression (Figure 3a to 3c), in bovine NP cells. FGF2 therefore plausibly enhances PG degradation in part through an upregulation of matrix-degrading enzymes.

Our sulfate incorporation results, however, suggest that the decrease in PG levels is at least in part due to decreased PG synthesis. We demonstrated an FGF2-mediated, dose-dependent suppression of PG synthesis as well as the inability of BMP7 to stimulate PG production in the presence of FGF2 in bovine disc cells. We therefore suggest that FGF2 exerts a dual effect on PG accumulation in spine discs via stimulation of PG degradation as well as inhibition of PG synthesis. Previous studies have demonstrated similar results in rabbit articular chondrocytes [35,36], in human OA cartilage [37], and in adult human articular chondrocytes [15], but this is the first study to do so in spine tissue.

Outside the joint, FGF2 is known to stimulate angiogenesis and, among other functions, play a role in wound repair [38-41]. It has also been shown to be a potent mitogen [35,42,43], and our results were consistent with this function. We found that FGF2 significantly stimulates proliferation of both NP and AF cells isolated from bovine tail IVD tissue (data not shown). Of note, we observed that FGF2 at concentrations of 1 and 10 ng/ml stimulates threefold and 16-fold induction of cell proliferation, respectively, compared with control (no FGF2 treatment) after 7 days. At a concentration of 100 ng/ml, we found >70-fold induction of NP cell proliferation after 21 days of incubation in alginate beads.

The mitogenic capabilities of FGF2 have sparked controversy over the exact role played by this growth factor in cartilage homeostasis. Previous studies have suggested that FGF2 acts as an anabolic mediator of cartilage homeostasis due to its mitogenic capacity, and several studies are currently using FGF2 in scaffolds for cartilage regeneration and repair [43-51] For example, FGF2 has been associated with a stimulation of cell proliferation in adult bovine articular cartilage [43,45] and in canine IVD cells [46]. Based on the results from this study as well as previous results from our laboratory [15,16], however, we suggest that the mitogenic effect of FGF2 in both human articular chondrocytes and bovine IVD tissue may be a pathologic sign of degeneration rather than regeneration. While FGF2 has already been found to substantially increase cell proliferation in bovine spine discs [52], it failed to increase ECM synthesis in parallel in our study, resulting in clustering of cells with little surrounding ECM – a hallmark of arthritic cartilage.

Further, we previously suggested that the increase in cell proliferation mediated by FGF2 in human articular cartilage may result from increased turnover of fibroblast-like cells rather than chondrocytes, resulting in fibrocartilage formation rather than the stronger, more durable hyaline cartilage [16]. We suggest the same principle in the IVD, as treatment of bovine NP cells with FGF2 stimulated an upregulation of collagen I compared with collagen II (data not shown), resulting in a decreased collagen II:I ratio and the formation of fibrocartilage compared with the collagen II-rich cartilage of a healthy IVD. Taken together, treatment of disc cells with FGF2 increases cell proliferation and decreases ECM production, resulting in clusters of disc cells in a fibrocartilage network similar to our findings from human articular cartilage [15].

Tsai and colleagues recently analyzed the effects of FGF2 on bovine NP cell growth and differentiation, and found that FGF2 stimulated increased sulfated PG synthesis, lowered aggrecan turnover, and lowered differentiation of the NP cell phenotype by maintaining responsiveness to TGFβ [53]. Our data, however, support the hypothesis that FGF2 serves primarily as an anti-anabolic factor rather than a pro-anabolic factor in cartilage homeostasis. Indeed, similar to results reported by Tsai and colleagues [53], we have found that FGF2 does stimulate an overall increase in sulfated PG synthesis. After normalizing these findings to cell number, however, our [^35^S]-sulfate incorporation and DMMB results suggest that, per cell, PG synthesis and total PG accumulation decreased dose dependently after treatment with FGF2. In addition, Tsai and colleagues reported increased gene expression of both collagen I and collagen II; however, we suggest that the ratio between type I and type II collagen may be more important than overall levels to determine the homeostatic effect in IVD tissue, and we have found an FGF2-mediated upregulation of collagen I compared with collagen II (data not shown), leading to the formation of a weak fibrocartilaginous network.

The potent mitogenic effect of FGF2 in cartilage has previously been correlated with FGF receptor activation. In the growth plate, for example, FGFR1 and FGFR3 have significant yet opposite roles in cartilage homeostasis. Binding of FGF2 to FGFR1 increases proliferation of chondrocytes, whereas binding of FGF2 to FGFR3 inhibits proliferation and therefore promotes differentiation [54-56]. The upregulation of FGFR1 with minimal expression of FGFR3 in the bovine IVD could therefore potentially explain the potent mitogenic effects of FGF2 in the spine disc. Interestingly, Valverde-Franco and colleagues found that, in the absence of signaling from FGFR3, a compensatory increase in interaction is seen between FGF2 and FGFR1, resulting in degradative effects such as defective articular cartilage with increased MMP-13 expression and increased cleavage products from type II collagen and aggrecan in mice [57].

Our studies revealed an upregulation of FGFR1 in degenerative disc tissue (Figure 1), as well as an FGF2-mediated increase of MMP-13 expression, but no FGF18-mediated effect on MMP-13 expression (Figure 3a to 3c). These results were similar to previous studies revealing that FGF18 acts primarily via FGFR3 in articular and growth plate cartilage [57,58]. We therefore suggest that FGF2, but not FGF18, utilizes FGFR1 to stimulate both mitogenic and anti-anabolic events in bovine spine IVD tissue. Further studies linking pathogenic disc degeneration and FGF-ligand binding activity to specific FGFRs may provide important information for understanding the potential role of FGFR1 in IVD homeostasis and disc degeneration.

Other studies have suggested an important role of FGF2 in the spontaneous resorption process of degenerative or herniated IVD tissue via stimulation of angiogenesis and/or inflammatory cytokines that aid in cartilage destruction [20,59,60]. Minamide and colleagues used a rabbit disc sequestration-type model to emulate IVD herniation *in vivo*, and found that epidural injection of FGF2 stimulates increased angiogenesis, increased speed of disc resorption, and increased the number of inflammatory cells compared with control (saline) [59]. Based on these findings, we suggest multiple roles of FGF2 in disc homeostasis depending on the stage of degeneration. In normal or recently injured disc tissue, FGF2 may act as an anti-anabolic mediator, suppressing PG synthesis and stimulating MMP-13 expression. These same properties, however, may be beneficial after disc herniation, stimulating degradation of herniated tissue and encouraging spontaneous disc resorption. The expression and role of FGF2 in different stages of degeneration should be further analyzed in human disc tissue, as well as in degenerative or herniated disc tissue, to gain a better understanding of its pathophysiologic function at each stage.

**Figure 8:**
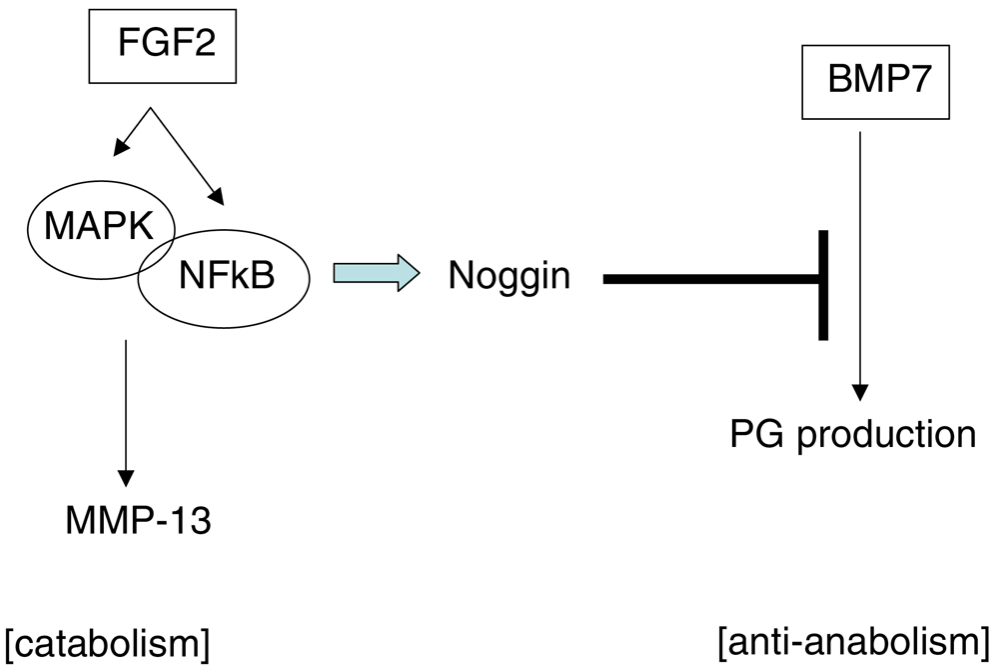
**Schematic of the regulation of catabolic and anti-anabolic actions of FGF2 in intervertebral disc cells**. Fibroblast growth factor 2 (FGF2) activates the mitogen-activated protein kinase (MAPK) and NF-κB pathways, which upregulate both MMP-13 and noggin gene expression. The upregulation of the noggin gene inhibits the anabolic transforming growth factor beta/bone morphogenetic protein signaling pathway, leading to decreased proteoglycan (PG) production.

Clinically, noggin may be a potential target for disc degeneration as it is a well-known inhibitor of the anabolic TGFβ/bone morphogenetic protein signaling pathway [31] and is upregulated by FGF2 in bovine disc tissue (Figure 7). Our pathway-specific inhibitor studies suggest that the ERK pathway is necessary for noggin stimulation by FGF2, while the NF-κB pathway (IKK) is involved in, but not necessary for, noggin stimulation leading to inhibition of BMP7 activity. These data suggest that mitogen-activated protein kinase (ERK) and NF-κB are involved in the anti-anabolic actions of FGF2, a factor that exerts its effects via multiple pathways (Figure 8). These results may be advantageous as pathway-specific inhibitors continue to gain favor as potential treatment strategies. Unlike treatment with FGF2, the stimulation of cells with IL-1β showed no significant increase in noggin expression, suggesting that the inhibitory effects of FGF2 and IL-1β on BMP7 are perhaps through distinct signaling pathways and biological actions.

## Conclusion

The present study suggests that the role of FGF2 can be defined as anti-anabolic and potentially catabolic in IVD cells. FGF2 enhances MMP-13, ADAMTS4, and ADAMTS5 expression at the transcriptional level, decreases PG synthesis, and inhibits the anabolic activity of BMP7-mediated PG synthesis. Moreover, it retains its mitogenic capacity in spine tissues while decreasing ECM formation, leading to clustering of cells often seen in arthritic states. The pathways involved are multiple and complex, and further investigation should be pursued to help gain a better understanding of the signaling cascades governing the interactions between FGF2, MMP-13 and BMP7.

## Abbreviations

ADAMTS = a disintegrin and metalloproteinase with thrombospondin motifs; AF = annulus fibrosus; BMP = bone morphogenetic protein; CM = cell-associated matrix; DMEM = Dulbecco's modified Eagle's medium; DMMB = dimethylethylene blue; ECM = extracellular matrix; FGF2 = fibroblast growth factor 2; FGFR = fibroblast growth factor receptor; IL = interleukin; IVD = intervertebral disc; MMP = matrix metalloprotease; NF = nuclear factor; NP = nucleus pulposus; PCR = polymerase chain reaction; PG = proteoglycan; RT = reverse transcriptase; TGFβ = transforming growth factor beta.

## Competing interests

The authors declare that they have no competing interests.

## Authors' contributions

H-JI participated in the study design, analysis and interpretation of data, manuscript preparation, and statistical analysis. XL participated in the study design, acquisition of data, analysis and interpretation of data, manuscript preparation, and statistical analysis. HSA and FP participated in the study design, collection of human tissue samples, analysis and interpretation of the data. ME participated in analysis and interpretation of the data, and manuscript preparation. EJT participated in the study design and manuscript preparation. DKP and RKU participated in the acquisition of tissues and helped data generation.
